# Supplementary material for: Cognitive stimulation in activities of daily living for individuals with mild-to-moderate dementia (CS-ADL): Study protocol for a randomised controlled trial
Source: PLoS One. 2024 Sep 3;19(9):e0309337. doi: 10.1371/journal.pone.0309337 (PMC11371241; doi:10.1371/journal.pone.0309337)
Supplement: S1 File — (DOCX) [file pone.0309337.s003.docx]

**Title:** Cognitive stimulation in activities of daily living for individuals with mild-to-moderate dementia: study protocol for a randomised controlled trial.

**Authors:** Simone Ryan, Dr. Orla Brady.

**Abstract**

*Background:* Multi-component CS programs incorporating practice of activities of daily living (ADL) into intervention have reported benefits for ADL outcomes in individuals living with mild-to-moderate dementia. A randomised controlled trial (RCT) within community occupational therapy services in Ireland, is planned to evaluate the effects of CS-ADL, an ADL-focused, multi-component CS program, on ADL outcomes for individuals living with mild-to-moderate dementia.

*Method:* A single-blind RCT with a calculated sample size of 34 participants has been planned to compare the effects of CS-ADL versus treatment as usual on the outcomes of basic ADLs and instrumental ADLs. Cognition, mood, communication, and quality of life will also be evaluated as secondary outcomes. CS-ADL sessions will run once weekly for a total of seven weeks, lasting approximately two hours each. Outcome data will be collected at baseline, within sessions and post-intervention at week eight. Descriptive statistics will be used to analyse the data.

*Discussion:*  CS programs are commonly conducted by occupational therapists working with individuals living with mild-to-moderate dementia. This study aims to demonstrate the effectiveness of a multi-component CS program delivered through an occupational therapy lens, potentially influencing the approach to CS and ADL interventions undertaken by occupational therapists.

**Introduction**

Dementia is a clinical syndrome characterised by deteriorating cognitive, behavioural and emotional functions that interfere with an individual’s performance in activities of daily living (ADL; American Psychiatric Association, 2013; Dolan and Shiel, 2017). The cognitive and social changes associated with the condition have been linked to decreased quality of life, increased caregiver burden, and higher care costs, contributing to dementia being one of the leading causes of hospitalisations and admissions to skilled nursing facilities worldwide (Laver et al., 2016). Dementia affects approximately 57.4 million people globally, with an estimated 53,932 individuals in Ireland living with dementia as of 2019 (Nichols et al., 2022; Pierse, Keogh and O’Neill, 2020). The widespread impact of dementia has prompted the development of a variety of treatments to slow the cognitive and functional decline associated with this condition (Arvanitakis, Shah & Bennett, 2019). Several non-pharmacological interventions are increasingly being used to mitigate this decline, with cognitive stimulation (CS) being one such approach.

CS is a non-pharmacological approach to intervention widely used with individuals with early-stage dementia. CS is typically delivered in a stimulating and rewarding environment, where participants engage in a range of activities and discussions aimed at enhancing global cognitive and social functioning, as opposed to a focus on specific functions in isolation (Clare and Woods, 2004). A variety of CS interventions have been described across the literature, including manualised therapies, individual programs, and CS delivered as part of a wider, multi-component intervention (Ryan & Brady, 2023). Cognitive stimulation therapy (CST) is one notable example of a manualised therapy commonly used by occupational therapists for people living with mild-to-moderate dementia. CST is a brief group intervention based on CS principles, consisting of 14 sessions of themed activities and discussions run over seven weeks (Spector et al., 2003). Several adaptations of this CST program have been developed and evaluated in the literature, including a 24-week maintenance program (MCST) and an individualised program (iCST). CST has demonstrated significant benefits for cognition, quality of life and communication for individuals with mild-to-moderate, as reported by Woods et al.’s (2012) systematic review. While it is assumed that cognitive improvements following CS interventions will transfer to everyday functioning, evidence from the literature does not support this assumption; no statistically significant effects of CS for ADL performance were reported (Woods et al., 2012).

However, Ryan & Brady’s (2023) scoping review exploring the use of CS in improving ADL outcomes for individuals with mild-to-moderate dementia mapped a variety of alternative CS programs that reported benefits for ADLs. Three randomised controlled trials (RCTs) with an overall total of 252 participants evaluated the effect of a German, multi-component group therapy termed MAKS (Graessel et al., 2011; Luttenberger et al., 2012a; 2012b). MAKS sessions consist of physical activities, individual/group cognitive stimulation exercises, and participation in ADL tasks, with all three studies reporting benefits of MAKS on ADL abilities. A cognitive-motor stimulation intervention (CMSI) was evaluated at 12 months by Olazarán et al. (2004) and at three years by Muñiz et al. (2015) through an RCT design. CMSI sessions consisted of individual/group cognitive exercises, psychomotor therapy, and ADL training. Favourable ADL outcomes were reported for the CMSI group when compared to a control. Furthermore, Jiménez Palomares et al.’s (2021) pilot RCT (*N=*58) investigating the effects of an occupational therapy CS program on ADL skills reported significant improvements in basic ADLs such as feeding, dressing and continence. Ryan and Brady’s (2023) scoping review observed a common trend across studies reporting positive effects for ADLs; multi-component interventions incorporating CS with ADL practice and/or physical activity produced more favourable ADL outcomes in comparison to traditional, discussion-based CS programs, like CST.

Occupational therapy is a profession where occupational performance and engagement in ADLs are central to intervention. While the nature of the scoping review methodology used by Ryan and Brady (2023) limits conclusions that can be drawn, the reduced evidence supporting the benefits of traditional CS programs for ADLs calls for a shift in approach used by occupational therapists. The incorporation of ADL-focused activities into multi-component CS programs demonstrate promising results for ADL outcomes (Graessel et al., 2011; Muñiz et al., 2015). However, the majority of multi-component CS interventions reported to benefit ADL outcomes by Ryan and Brady (2023) recruited participants from nursing homes or long-term care facilities. The lack of data evaluating the implementation of these programs within the community setting prompted the development of CS-ADL, a multi-component group CS program that aims to enhance ADL performance alongside social and cognitive functioning.

The CS-ADL program was scientifically developed based on the currently available literature evidence and appropriately revised after it was successfully piloted in an Irish, community-based Psychiatry of Later Life service. Previous qualitative research has explored the experience of taking part in CS-ADL, reporting that both participants and their caregivers perceive CS-ADL to be an acceptable intervention that positively influenced the daily lives of both dyad members with benefits reported in the memory, mood and social interaction of participants (Ryan, Chockalingam & Brady, 2023). However, CS-ADL is a newly developed intervention; no previous studies have explored the effectiveness of CS-ADL on improving ADL outcomes. Therefore, an RCT evaluating the effect of CS-ADL on ADL outcomes when compared to a group receiving treatment as usual (TAU) is proposed. This RCT will take place in Irish community occupational therapy services, and individuals living with mild-to-moderate dementia will be recruited to take part.

*Aims and objectives*

The aim of this study is to evaluate the effect of CS-ADL, an ADL-focused multi-component CS program, on ADL outcomes for people living with mild-to-moderate dementia. The primary objective of this study is to determine the effect of CS-ADL on basic ADL (BADL) and instrumental ADL outcomes (IADL). The secondary objectives of the study include examining the effects of the intervention on cognition, mood, quality of life (QOL) and communication. These secondary outcomes were chosen as previous CS programs have demonstrated benefits for these variables (Woods et al., 2012).

*Hypotheses*

*Null hypotheses*

CS-ADL does not benefit ADL outcomes in people with mild-to-moderate dementia when compared to TAU.

CS-ADL does not benefit cognition, mood, quality of life and communication in people with mild-to-moderate dementia when compared to TAU.

H_0_: µ_CS-ADL_ = µ_TAU_

*Alternate hypotheses*

CS-ADL benefits ADL outcomes in people with mild-to-moderate dementia when compared to TAU.

CS-ADL benefits cognition, mood, quality of life and communication in people with mild-to-moderate dementia when compared to TAU.

H_1_: µ_CS-ADL_ > µ_TAU_

**Materials and methods**

*Trial design*

This will be a multi-centre, parallel, single-blind, randomised superiority trial of CS-ADL versus TAU for people with mild-to-moderate dementia. Sample size was calculated using formal power analysis with G*Power software (Faul et al., 2007). Based on Cohen’s (1988) guidelines for small (*r =* 0.1), medium (*r* = 0.3) and large (*r =* 0.5) effects for a 2 (pre- and post-test) x 2 (CS-ADL and TAU) *t*-test, with the difference between two dependent means (matched pairs), sample size was calculated for an estimated small (*d =* 0.2), medium (*d* = 0.5) and large (*d* = 0.8) effect size. Two-tailed alpha of 0.05 was assumed for all tests. With an estimated effect size of 0.2, a sample size of 199 is required for a critical t of 1.9720175 and degrees of freedom (df) of 198, with an actual power of 0.8016910. With an estimated effect size of 0.5, a sample size of 34 is required for a critical t of 2.0345153 and a df of 33 and an actual power of 0.8077775. For an estimated effect size of 0.8, a sample size of 15 was calculated to be required for a critical t of 2.1447867 and a df of 14, with an actual power of 0.8213105. It is expected there will be challenges in the recruitment of 199 participant for the estimated effect size of 0.2, therefore for pragmatic reasons, recruitment will aim for the sample size of 34 (*d* = 0.5). Participants will be randomised with 1:1 allocation ratio to either the intervention or control group. The primary and secondary outcomes will be measured at baseline prior to randomisation and at the end of intervention (8 weeks). There will also be an observational assessment of occupational performance completed by the treating therapists within every group session.

*Study setting*

The study will take place in two Irish Health Service Executive (HSE), community-based occupational therapy services. The principal investigator will deliver the CS-ADL intervention.

*Eligibility criteria*

Eligibility criteria for this study reflects criteria of previous multi-component CS interventions. Participants must have a formal diagnosis of major neurocognitive disorder (dementia) as per the DSM-V criteria (American Psychiatric Association, 2013). Participants must have a mild-to-moderate cognitive impairment as classified by the Mini Mental State Examination (MMSE) or equivalent screens (Folstein et al., 1975). Individuals with severe dementia, i.e., an MMSE<10, will be excluded as most CS interventions are not applicable for those with severe cognitive impairment (Spector et al., 2003). Participants taking dementia medication can continue to do so during the study.

Participants must have some ability to communicate and understand communication, determined by a score of 1 or 0 on questions 12 and 13 of the Clifton Assessment Procedures for the Elderly-Behaviour Rating Scale (Pattie and Gilleard, 1979). Participants must be able to see and hear well enough to participate in the group. Participants will not be excluded if they have significant uncontrolled disruptive behaviours, a premorbid diagnosis of a learning disability, or a significant physical illness/disability that may affect participation during intervention sessions or assessments.

*Recruitment*

Participants will be recruited from HSE occupational therapy settings in Ireland. Clinicians will be encouraged to screen their case load for potential participants and approach them and their caregiver to discuss the study. If they are interested in taking part, internal referral protocols will be followed. Contact details will be passed to the research team who will arrange a meeting with the participant and their caregiver to assess eligibility. If they are eligible and agree to take part, informed written consent will be obtained. Participants’ capacity to consent to take part in the study will be assessed following the guidelines stipulated in the Assisted Decision-Making (Capacity) Act (2015). If the participant with dementia lacks the capacity to consent, appropriate protocols will be adhered to in line with the Assisted Decision-Making (Capacity) Act (2015) which is currently evolving into full enactment in Ireland.

*Randomisation and masking*

Randomisation will occur after eligibility, consent and baseline assessments have been carried out. Random allocation will be completed by an allocator otherwise uninvolved in the study. A computer-generated number system (RANDBETWEEN Command on Microsoft Excel) will be used. While participants cannot be blinded to their allocated group, outcome assessors will be blind to group allocation.

**Intervention**

*CS-ADL*

The intervention group will receive a program of CS-ADL, with sessions delivered once weekly across 7 weeks. Typical CST programs commonly delivered by Irish occupational therapy services span a duration of 7 weeks, demonstrating that an intervention of this length is feasible within these services. CS-ADL sessions will last approximately 2 hours. While Ryan and Brady’s (2023) scoping review reported significant ADL benefits from the intensive multi-component CS interventions of Graessel et al. (2011) and Muñiz et al. (2015), Jiménez Palomares et al.’s (2021) occupational therapy CS program reported benefits for ADLs through a less intensive approach of two 45-minute sessions per week for a total of 5 weeks. As typical community-based CST programs often deliver two 45-minute sessions on the one day to aid feasibility, the planned intensity and duration of the CS-ADL program is therefore justified. At least one qualified occupational therapist will facilitate the sessions, with the assistance of a co-facilitator, as CS-ADL activities require a level of supervision not feasible for a single facilitator. Furthermore, the lead group facilitator must be an occupational therapist as the aims of CS-ADL align with the core principles of the profession. Each session consists of similar components, however the sequence and timing allocated per component may differ between sessions. Activities are based around a different theme for each session, for example morning routine, evening routine, or domestic activities, with each session intended to be enjoyable and mentally stimulating for the individual. A typical session will begin with 20-minutes of introductions, reality orientation, a group song and discussion of the news of the week. 15-minutes will then be spent on a physical activity/game. Physical activity will be a key component of the CS-ADL program as 7 of 9 CS programs reviewed by Ryan and Brady (2023) involving a physical activity component to intervention reported benefits for ADL outcomes. A physical activity at the start of the group will also aim to benefit arousal levels of participants and build rapport amongst group members. This will be followed by 25-minutes of ADL-focused cognitive stimulation activities, a 15-minute break, and approximately 10 more minutes of ADL-focused CS activities. These activities will include the identification and categorisation of everyday items, discussion, reminiscence, planning and sequencing of ADLs and the completion of everyday writing/calculation tasks. This will be followed by a 30-minute ADL task, which is an essential component to the program, as Ryan and Brady’s (2023) review found CS interventions with an ADL component report significant benefits for ADL outcomes. Examples of activities will include making a breakfast, a simple gardening activity or sorting laundry. While the structure of CS-ADL has taken inspiration from previous CS programs (Graessel et al., 2011; Jiménez Palomares et al., 2021; Spector et al., 2003), CS-ADL differs as participants are actively engaged in the planning, preparation, and practice of everyday activities like washing, dressing, and cooking. A full groupwork protocol and detailed session plans are available at Appendix 1 and Appendix 2.1 respectively. Sample handouts and instructional cards used in the implementation of CS-ADL sessions are available from appendix 2.2 to appendix 2.8. Therapists involved in the administration of CS-ADL are advised to use their clinical judgement in the planning and implementation of sessions, as sessions should be individualised to suit the needs, abilities, and interests of group members. Activities should be adapted accordingly.

*Treatment-as-usual*

The control group will continue to have access to treatment as usual, which will include medication, input from health professionals and any activities provided in the day hospital if the participant is in attendance. After the completion of the study, participants and their caregivers will be offered a home programme based on the CS-ADL intervention and will be given training in how to use it.

*Outcome measures*

Outcome measures will be recorded in both CS-ADL and TAU participants at baseline (week 0) and post-intervention (week 8). The completion of outcome measures pre- and post-intervention will be mandatory for participation in the trial; participants and caregivers will be reminded of requirements prior to the commencement of the group and after the completion. Socio-demographic information of participants will be collected including age, gender, ethnicity, diagnosis, and medication.

*Primary outcomes: BADL and IADL*

Change in both BADL and IADL will be measured using the Alzheimer's Disease Cooperative Study-Activities of Daily Living (ADCS-ADL) scale (Galasko et al., 1997). This scale was designed for the assessment of ADL outcomes for individuals with Alzheimer's disease in clinical trials. The scale consists of 23 items that includes 6 BADL and 17 IADL outcomes, scored on a range from 0 (patient does not perform the activity), to the highest score (patient is independent in the activity). The scale is administered with caregivers who are asked to rate the degree to which their care-recipient performs each item in the last four weeks (Custodio et al., 2022). The ADCS-ADL has been identified to be a reliable and valid instrument, with high internal consistency and sensitivity to functional changes in individuals with mild-to-moderate dementia (Rózsa et al., 2009).

*Secondary outcomes: Cognition, communication, QOL, mood.*

General cognitive functioning will be measured using either the Montreal Cognitive Assessment (MoCA) face-to-face or the MoCA-Blind delivered via the telephone, dependent on feasibility for participants and assessors. The MoCA is a brief screening tool used to detect cognitive impairment and consists of a 30-point test assessing the cognitive domains of visuospatial/executive, naming, memory, attention, language, abstraction, delayed recall, and orientation. The MoCA exhibits high sensitivity and specificity and its validity for use with people living with dementia (PLwD) has been well established (Nasreddine et al., 2005). The MoCA-Blind does not require visual input and was developed to enable cognitive screening of those with visual impairment, also enabling administration through telephone or online formats. The MoCA-Blind is scored on a scale of 22 points as visual elements are excluded. While specificity remains on par with the original MoCA, sensitivity is reduced (Wittich et al., 2010).

Cognition will be assessed using the Repeatable Battery for Assessment of Neuropsychological Status (RBANS; Randolph, 1998). The RBANS is a commonly used brief battery of cognitive function for individuals with dementia. This battery consists of 12 subtests which assess five cognitive domains: immediate memory, visuospatial-constructional ability, language and delayed memory. This battery has demonstrated good reliability and adequate validity indicators (Shura et al., 2018).

Communication ability will be assessed using the Holden Communication Scale (HCS; Holden and Woods, 1995). This scale includes 12 items assessing the domains of conversation, awareness, humour, and responsiveness. Each items contains five response options ranging from 0 to 4. Total scores can range from 0 to 48 with higher scores indicating more communication difficulties. The HCS was initially developed to assess communication outcomes in reality orientation and reminiscence programs and has demonstrated good reliability and validity for use with PLwD (Strøm et al., 2016). While there are limitations to reliability and validity data outside of the original development of the tool (Dolan and Shiel, 2017), the HCS has been used in various CS studies to assess communication (e.g., Orrell et al., 2005; Spector et al., 2003).

QOL will be assessed using the Quality of Life-Alzheimer’s Disease Scale (QOL-AD). This scale consists of 13 items spanning the domains of physical health, energy, mood, living situation, memory, family, marriage, friends, chores, fun, money, self, and life as a whole. Response options include 1 (poor), 2 (fair), 3 (good) and 4 (excellent), with total scores ranging from 13-52. Assessment is delivered through an interview format and separate ratings are obtained from the participant themselves and the caregiver. Higher scores indicate a better quality of life. The QOL-AD has demonstrated good reliability and internal consistency (Logsden et al., 1999) and has been found to detect improvements in QOL in previous CS studies (Orrell et al., 2014; Spector et al., 2003).

Mood will be assessed through the presence of neuropsychiatric symptoms as measured by the Neuropsychiatric Inventory Questionnaire (NPI-Q), a version of the Neuropsychiatric Inventory (NPI; Kaufer et al., 1998). The NPI-Q is a caregiver-based questionnaire that measures the presence and severity of 12 neuropsychiatric symptoms in PLwD, including delusions, hallucinations, apathy, disinhibition, and agitation/aggression. The severity scale runs from 1 to 3 points (1=mild, 2=moderate, 3=severe). The questionnaire also measures the respective level of caregiver distress associated with each symptom, with the scale running from 0 to 5 points (0=no distress, 5=extreme distress). The scale is widely used in research, has demonstrated acceptable levels of internal consistency, and has been cross validated with the standard NPI in clinical practice settings (Cummings, 1994).

*Other outcome measures*

Occupational performance within a group setting will be measured after each session using the Occupational Therapy Task Observation Scale (OTTOS). The OTTOS contains two parts, with 10 items for evaluation of specific task functions and 5 items for rating general behaviour. The reliability and validity of the OTTOS has been demonstrated (Margolis et al., 1996).

*Statistical analysis*

The study sample will be analysed using descriptive statistics through the latest version of Statistical Package for Social Sciences (SPSS). Data on recruitment will be recorded and examined. A Consolidated Standards of Reporting Trials (CONSORT) diagram describing the flow of participants through the study will be presented in the final RCT report, detailing enrolment, allocation, follow-up, and analysis of data (Figure 1). Baseline data will be assessed for normality and if normally distributed, parametric analysis will be used. Where data is not normally distributed, non-parametric analysis will be used. Types of tests chosen will be based on whether the data is parametric or non-parametric. Significance levels will be set to 0.05. A linear regression model adjusting for baseline scores will be used to estimate the effect of intervention on each of the primary and secondary outcomes, with a between-group analysis completed to examine if the groups are significantly different at outcome. A within-group analysis will be also completed to evaluate if there are any significant changes within groups. Sociodemographic factors like age and gender will be entered as covariates.

*Ethical considerations*

The researchers are currently in the process of receiving ethical approval for this study.

*Anticipated risks for trial participants*

There appear to be no documented harmful side effects from participating in typical CS programs, with no adverse reactions apparent. Health and safety risks, including the risk of falls may be posed by the participation in cooking tasks and physical activities. However, group facilitators will use their clinical judgement, clinical risk assessments in the implementation of activities and always ensure adequate supervision is given. Nevertheless, potential participants and their caregivers will be fully informed of the potential risks before partaking in the trial.

**Discussion**

Dementia is a progressive syndrome where deteriorating cognitive, behavioural, and emotional functions impede on the individual’s ability to engage in meaningful occupations and ADLs. Traditional discussion-based CS programs have not demonstrated consistent benefits for ADL outcomes for individuals with mild-to-moderate dementia. This has prompted the development of CS-ADL, an ADL-focused multi-component CS program, informed by the results of Ryan and Brady’s (2023) scoping review and a pilot study conducted within the Mullingar PLL services. A single-blind, parallel, randomised superiority trial has been proposed, aiming to evaluate the benefits of CS-ADL on the ADL outcomes of individuals living with mild-to-moderate dementia, in comparison to TAU. The proposed RCT also intends to evaluate the effects of CS-ADL on cognition, communication, QOL and mood of participants in comparison to TAU. To the extent of the authors knowledge, this study will be the first of its kind conducted within the Irish PLL services and will provide valuable evidence of the effectiveness and feasibility of implementation of a multi-component CS intervention in a community setting. This study aims to influence the approach to CS intervention undertaken by Irish occupational therapists working in community PLL services.

Funding: Funding for this trial will be sought from the Irish Research Council, Alzheimer's society of Ireland or the Health Research Board, Ireland. Funding will contribute to the provision of materials for the CS ADL sessions and the transport of participants to the study centre.

Contributors: Simone Ryan is the primary investigator for this study. Dr. Orla Brady is the co-investigator of the study and is responsible for the original concept of the trial and for ensuring that the study follows the agreed protocol. Martina Logan, Sophie Lynch, staff grade occupational therapists, and Simone Ryan were involved in the design of the study’s group protocol and for piloting the intervention sessions. OB and SR were involved in the review and approval of the RCT protocol.

**References**

American Psychiatric Association (2013) Diagnostic and Statistical Manual of Mental Disorders (5^th^ edn.). Arlington, VA: American Psychiatric Association.

Arvanitakis Z, Shah RC and Bennett DA (2019) Diagnosis and Management of Dementia: A Review. *JAMA* 322(16): 1589-1599.

Clare L and Woods RT (2004) Cognitive training and cognitive rehabilitation for people with early-stage Alzheimer’s disease: A review. *Neuropsychological Rehabilitation* 14(4): 385-401.

Cohen J (1988) Statistical Power Analysis for the Behavioural Sciences (2^nd^ edn.). Hillsdale, NJ: Erlbaum.

Cummings J, Mega M, Gray K, et al. (1994) The Neuropsychiatric Inventory: Comprehensive assessment of psychopathology in dementia. *Neurology* 44: 2308-2314.

Dolan O and Shiel A (2017) Rehabilitation approaches in dementia: an evaluation of three interventions from an occupational therapy perspective. PhD Thesis, NUI Galway, Ireland.

Faul F, Erdfelder E, Lang AG, et al. (2007) G*Power 3 : a flexible statistical power analysis for the social, behavioural, and biomedical sciences. *Behaviour Research Methods* 39: 175-191.

Folstein MF, Folstein SE and McHugh PR (1975) Mini-mental state. A practical method for grading the cognitive status of patients for the clinician. *J Psychiatr Res* 12(3): 189-198.

Graessel E, Stemmer R, Eichenseer B, et al. (2011) Non-pharmacological, multi-component group therapy in patients with degenerative dementia: a 12-month randomised, controlled trial. *BMC Medicine* 9(129): 1-11.

Graf C (2008) The Lawton Instrumental Activities of Daily Living (IADL) Scale. *AJN* 108(4) : 59.

Holden UP and Woods RT (1995) Positive Approaches to Dementia Care (3^rd^ edn.). Edinburgh: Churchill Livingstone.

Jiménez Palomares M, González López-Arza MV, Garrido Ardila EM et al. (2021) Effects of a Cognitive Rehabilitation Programme on the Independence Performing Activities of Daily Living of Persons with Dementia- A Pilot Randomised Controlled Trial. *Brain Sciences* 11(3): 319.

Kaufer DI, Cummings JL, Christine D, et al. (1998) The impact of neuropsychiatric symptoms in Alzheimer’s disease: the Neuropsychiatric Inventory Caregiver Distress Scale. *Journal of the American Geriatrics Society* 46: 210-216.

Laver K, Suzanne D, Whitehead C et al. (2016) Interventions to delay functional decline in people with dementia: a systematic review of systematic reviews. *BMJ Open* 6(4): e010767.

Lawton MP and Brody EM (1969) Assessment of Older People: Self-Maintaining and Instrumental Activities of Daily Living. *The Gerontologist* 9(3): 179-186.

Logsdon RG, Gibbons LE, McCurry SM, et al. (1999) Quality of life in Alzheimer’s disease: Patient and caregiver reports. *Journal of Mental Health and Aging* 5(1): 21-32.

Luttenberger K, Donath C, Uter W, et al. (2012a) Effects of Multimodal Nondrug Therapy on Dementia Symptoms and Need for Care in Nursing Home Residents with Degenerative Dementia: A Randomised-Controlled Study with 6-Month Follow-up. *JAGS* 60(5):830-840.

Luttenberger K, Hofner B and Graessel E (2012b) Are the effects of a non-drug multimodal activation therapy of dementia sustainable? Follow-up study 10 months after completion of a randomised controlled trial. *BMC Neurology* 12(151): 1-9.

Mahoney FI and Barthel DW (1965) Functional evaluation: The Barthel Index. *Md State Med J.* 14:61-65.

Margolis RL, Harrison SA, Robinson HJ, et al. (1996) Occupational therapy task observation scale (OTTOS): a rapid method for rating task group function of psychiatric patients. *American Journal of Occupational Therapy* 50(5): 380-385.

Muñiz R, Serra CM, Reisberg B, et al. (2015) Cognitive-Motor Intervention in Alzheimer’s Disease: Long-Term Results from the Maria Wolff Trial. *Journal of Alzheimer’s Disease* 45:295-304.

Nasreddine ZS, Phillips, NA, Bédirian V, et al. (2005) The Montreal Cognitive Assessment, MoCA: A Brief Screening Tool for Mild Cognitive Impairment. *Journal of the American Geriatric Society* 53(4): 695-699.

Nichols, E et al. (2022) Estimation of the global prevalence of dementia in 2019 and forecasted prevalence in 2050: An analysis for the Global Burden of Disease Study 2019. *The Lancet Public Health* 7(2): 105-125.

Office of the Attorney General. *Assisted Decision-Making (Capacity) Act 2015,* no 65 of 2015.

Orrell M, Spector A, Thorgrimsen L, et al. (2005) A pilot study examining the effectiveness of maintenance Cognitive Stimulation Therapy (MCST) for people with dementia. *International Journal of Geriatric Psychiatry* 20: 446-451.

Olazarán J, Muñiz R, Reisberg B, et al. (2004) Benefits of cognitive-motor intervention in MCI and mild to moderate Alzheimer disease. *Neurology* 63(12): 2348-2353.

Pattie AH and Gilleard CJ (1979) Manual of the Clifton Assessment Procedures for the Elderly (CAPE). Sevenoaks: Hodder and Stoughton.

Pierse T, Keogh F and O’Neill S (2020) Generating national projections of dementia cases for Ireland using a calibrated macro-stimulation model. *BMJ Open* 10(8): e035463.

Pietra GLD, Savio K, Oddone E, et al. (2011) Validity and reliability of the Barthel index administered by telephone. *Stroke* 42(7): 2077-2079.

Ryan S and Brady O (2023) Cognitive stimulation and activities of daily living for individuals with mild-to-moderate dementia: a scoping review. British Journal of Occupational Therapy. 2023;0(0). doi:10.1177/03080226231156517

Schulz KF, Altman DG, Moher D, for the CONSORT Group (2010) CONSORT 2010 Statement: updated guidelines for reporting parallel group randomised trials. *J Clin Epi*  63(8): 834-840.

Spector A, Thorgrimsen L, Woods B et al. (2003) Efficacy of an evidence-based cognitive stimulation therapy programme for people with dementia: Randomised controlled trial. *British Journal of Psychiatry* 183(3): 248-254.

Strøm BS, Engedal K, Benth JS, et al. (2016) Psychometric evaluation of the Holden Communication Scale (HCS) for persons with dementia. *BMJ Open* 6(12): e013447

Wittich W, Phillips N, Nasreddine ZS, et al. (2019) Sensitivity and Specificity of the Montreal Cognitive Assessment Modified for Individuals who are Visually Impaired. *Journal of Visual Impairment and Blindness* 104(6): 360-368.

Woods B, Aguirre E, Spector AE et al. (2012) Cognitive stimulation to improve cognitive functioning in people with dementia. *Cochrane Database of Systematic Reviews* (2): 1-72.

Appendix 1 CS-ADL Groupwork Protocol

| **CS-ADL Groupwork protocol** | |
| --- | --- |
| **Group title** | Cognitive stimulation in activities of daily living for individuals with mild-to-moderate dementia (CS-ADL) |
| **Author(s)** | Dr. Orla Dolan, senior occupational therapist; lecturer in Occupational Therapy.  Simone Ryan, occupational therapist and masters of philosophy (M.Phil) student in occupational therapy, University of Galway.  Martina Logan, staff grade occupational therapist.  Sophie Lynch, staff grade occupational therapist. |
| **How to reference:** | Dolan, O., Ryan, S., Logan, M., Lynch, S. (2022) Cognitive stimulation in activities of daily living for individuals with mild-to-moderate dementia (CS-ADL). [Unpublished manuscript]. |
| **Purpose** | This group aims to provide benefits for the performance of activities of daily living (ADLs) for individuals with mild-to-moderate dementia. This group involves the delivery of cognitive stimulation through an occupational therapy lens; physical activity and ADL-based activities are incorporated into a program of cognitive stimulation, with the aim to improve/maintain ADL outcomes. |
| **Evidence-base** | Cognitive stimulation (CS) involves engagement in a range of activities and discussions with the aim to enhance cognitive and social functioning (Clare and Woods, 2004). However, a recent scoping review of the use of CS in ADLs for individuals with mild-to-moderate dementia reported that traditional, discussion-based CS approaches do not benefit ADL outcomes (Ryan and Dolan, 2022). In comparison, studies evaluating multi-component approaches to CS, incorporating physical activity and ADL-based activities into their program reported significant benefits for ADLs (Graessel et al., 2011; Jiménez Palomares et al., 2022). |
| **Frames of reference** | Activities will be based on a rehabilitative frame of reference (FoR). This FoR is typically used with individuals whose underlying difficulties are unlikely to be remediated and will therefore be used to inform this group as dementia is often of a progressive, degenerative nature (Gale et al., 2018). The rehabilitative FoR aims to maximise independence by utilising areas of strength rather than attempting to remediate areas of deficit. This will be achieved using cognitive stimulation strategies and ADL practice in conjunction with the instruction of compensatory methods like assistive equipment and memory aids. Members will also gain support from each other through group discussion and problem-solving of everyday scenarios. |
| **Logistics** | *Place of meeting:* Occupational therapy services. Group room to have adequate table and seating arrangements.  *Number of clients:* A closed group of approximately 5-10 individuals.  *Group facilitators:* The group will be facilitated by at least one qualified occupational therapist. Co-facilitation can be by fellow occupational therapist, healthcare assistant, student or relevant health and social care professional.  *Schedule:* The program will span 7 weeks, consisting of once weekly sessions lasting approximately 2 hours.  *Advertisement/promotion:* The group will be promoted to fellow clinicians within local services, who will screen their caseload for potential participants. |
| **Group structure** | Each session consists of similar components, however the sequence and timing allocated per component may occasionally differ between sessions. An example of the session structure is detailed below:  Sessions will begin with introductions, reality orientation, a group song, and a discussion of news of the week. This will be followed by warm-up exercises before a physical activity/game. ADL-focused cognitive stimulation activities will be completed before a 15-minute break, after which the main ADL-based activity will be completed. Sessions will be concluded with a group song. A full breakdown and method of each session is detailed below. |
| **Group goals** | This group aims to:  -Provide opportunity for participants to receive peer support for memory and thinking challenges and/or the experiences of later life through a group environment.  -Provide a supportive environment in which participants with communication difficulties can express themselves amongst their peers.  -Provide benefits for cognition including improving the arousal levels of participants.  -Provide health benefits of exercise for participants through engagement in low-impact physical activity.  -Benefit mood of participants through opportunities to engage in physical activity, socialisation, and meaningful occupations.  -Provide an environment where participants are actively engaged and involved in the planning, preparation, and practice of ADLs.  -Increase participants confidence in the performance of ADLs.  -Introduce compensatory approaches to ADLs, providing opportunities for participants to explore compensatory devices and techniques of interest to them. |
| **Essential requirements for group membership** | -Older adults with a diagnosis of major neurocognitive disorder as per the DSM-V criteria (American Psychiatric Association, 2013).  -Group members must have a mild-to-moderate cognitive impairment, as assessed by the MoCA, or MoCA-Blind administered over the telephone. Individuals with severe cognitive impairment will not be suitable for group membership.  -Group members must have some ability to communicate and understand communication, determined by a score of 1 or 0 on questions 12 and 13 of the Clifton Assessment Procedures for the Elderly-Behaviour Rating Scale (Pattie and Gilleard, 1979).  -Individuals with significant uncontrolled disruptive behaviours, a premorbid diagnosis of learning disability, or a significant physical illness/disability that affects participation during intervention sessions will not be suitable for group membership. |
| **Outcome measures** | Primary outcomes of the group will be basic ADL (BADL) and instrumental ADL (IADL) outcomes.  -The Alzheimer’s Disease Co-operative Study – Activities of Daily Living (ADCS-ADL) will be used to measure BADL and IADL outcomes.  Secondary outcomes will be cognition, communication, mood, and quality of life.  -Cognition is a secondary outcome of this group and general cognitive functioning will be measured using the Montreal Cognitive Assessment (MoCA) or MoCA-Blind via telephone, dependent on feasibility for participants or the assessor. Cognitive functioning will be assessed using the Repeatable Battery for Assessment of Neuropsychological Status (RBANS). This battery assesses five cognitive domains: immediate memory, visuospatial-constructional ability, language and delayed memory.  -Communication ability will be measured using the Holden Communication Scale (HCS).  -Quality of life will be assessed using the Quality of Life-Alzheimer’s Disease Scale (QOL-AD).  -Mood will be assessed through the presence of neuropsychiatric symptoms as measured by the neuropsychiatric inventory questionnaire (NPI-Q). |
| **Referral criteria** | Please refer to eligibility criteria. Local occupational therapy referral protocols to be followed. |
| **References** | American Psychiatric Association (2013) Diagnostic and Statistical Manual of Mental Disorders (5th edn.). Arlington, VA: American Psychiatric Association.  Clare L and Woods RT (2004) Cognitive training and cognitive rehabilitation for people with early-stage Alzheimer's disease: A review. *Neuropsychological Rehabilitation* 14(4):385-401.  Gale SA, Diler A, Daffne KR (2018) Dementia. *The American Journal of Medicine* 131(10):1161-1169.  Graessel E, Stemmer R, Eichenseer B, et al. (2011) Non-pharmacological, multicomponent group therapy in patients with degenerative dementia: a 12-month randomised, controlled trial. *BMC Medicine* 9(129): 1-11.  Jiménez Palomares M, González López-Arza MV, Garrido Ardila EM et al. (2021) Effects of a Cognitive Rehabilitation Programme on the Independence Performing Activities of Daily Living of Persons with Dementia-A Pilot Randomised Controlled Trial. *Brain Sciences* 11(3): 319.  Lawton MP and Brody EM (1969) Assessment of Older People: Self-Maintaining and Instrumental Activities of Daily Living. *The Gerontologist* 9(3):179-186.  Mahoney FI and Barthel DW (1965) Functional evaluation: The Barthel Index. *Md State Med J.* 14:61-65.  Pattie AH and Gilleard, CJ (1979), Manual of the Clifton Assessment Procedures for the Elderly (CAPE), Hodder and Stoughton, Sevenoaks.  Ryan S and Dolan O (2022) Cognitive Stimulation and Activities of Daily Living for Individuals with Mild-to-Moderate Dementia: A Scoping Review. [Manuscript submitted for publication]. |

Appendix 2.1 CS-ADL individual session plans

| **Session Number:** | 1 of 7 |
| --- | --- |
| **Group Title:** | Cognitive stimulation in activities of daily living for individuals with mild-to-moderate dementia (CS-ADL) |
| **Session Theme:** | Morning routine |
| **Preparation:** | |
| *Set-up* | Arrange adequate seats around a table in the centre of the room. Place a newspaper on the table. Ensure a whiteboard/flipchart and a clock are set up and visible in the room. Set up a separate cooking station near an outlet with hotplates available on the table (if a hob is not already available in the room). Ensure all other materials necessary are available in the room. |
| *Materials* | Whiteboard/flipchart, marker, newspaper, inflatable ball, fly swatters and balloon, 8 drawstring bags, 2-4 cardboard boxes, assortment of cleaning, cooking, and grooming equipment e.g., deodorant, toothpaste, salt and pepper shakers. Laminated instruction cards: ‘getting ready for the day’, ‘cleaning’, ‘breakfast’. Array of assistive devices e.g., button hook, shoehorn, pillbox. Chopping boards, knives and forks, hotplates, frying pans, spatula, take-away plates, and tinfoil. Breakfast foods e.g., eggs, sausages, bacon, tomatoes. |
| **Format:** | |
| *Introduction* (20 minutes) | Greetings.  Warm-up activity: Pass inflatable ball/balloon around the group. Instructions provided to introduce self while holding the ball and to tell the group about themselves.  Group provides suggestions of potential group names and a song that will be sung at the beginning of each session. (If necessary, group facilitators should have a variety of options for group members to choose from). Group name to be written on whiteboard/flipchart.  Orientation of day of the week, date, month, year, season, and time to be discussed and written on whiteboard.  Discussion of news of the week. |
| *Physical activity* (15 minutes) | Gentle seated stretches to warm-up.  Seated physical activity to involve a team balloon game. This game involves keeping a balloon up in the air and off the floor by using fly swatters. Group members should be split into two teams and seated opposite to each other. Group members should work in their teams to try and pass the balloon to a goal without letting the balloon fall to the ground. Group facilitators can act as the ‘goal’. |
| *Cognitive stimulation activities* (30 minutes) | Perception-based activities: group members asked to place their hand into a bag without looking and identify everyday items relating to morning routine through touch alone. Prompting questions to be provided by group facilitators, members to verbally describe what they would do with the item.  Group members to break off into smaller groups if possible. Members to rummage through cardboard boxes filled with an assortment of cleaning, cooking and grooming items related to the theme of morning routine. Members to sort items in the box into categories by placing them on laminated instruction cards of ‘cleaning’, ‘breakfast’, and ‘getting ready for the day’ (appendix 2.2). Members encouraged to discuss, and problem solve together.  An array of assistive devices related to the theme of morning routine to be placed in a bag e.g., shoehorn, button hook, jar opener, pillbox. Group members to pull out an item from the bag and guess its purpose. Use of assistive devices to be demonstrated by group facilitators with practice of use by group members. |
| *Break* (15 minutes) | Tea/coffee and biscuits to be provided to group members. Group facilitators to encourage casual conversation amongst members. |
| *Cognitive stimulation activities* (10 minutes) | Discussion and sequencing of morning routine amongst group members: getting ready for the day, breakfast routine. Discussion and reminiscence of various breakfast recipes. |
| *ADL practice* (30 minutes) | Group members to split into smaller groups and asked to prepare and cook a fried breakfast from a range of ingredients, using the hob and pans. Recipe discussion and reminiscence to be encouraged throughout by group facilitators. Food to be served up onto take-away plate to take home. |
| *Conclusion* (5 minutes) | Group facilitators to summarise session and ask for feedback. Group to sing group song. Facilitators to remind everyone of next session. |
| **Rationale for activities chosen:**  A physical game was chosen as the first activity to build rapport amongst members and facilitate socialisation within the group. Physical activity was also a component of a variety of CS approaches reviewed by Ryan and Dolan (2022), with 7 of 9 studies involving a physical activity component to intervention reporting benefits for ADL outcomes.  Chosen activities aim to target a variety of cognitive domains. Targeted domains of this group are reflective of previous CS programmes reported to have ADL benefits (Jiménez Palomares et al., 2022; Muñiz et al., 2015). Orientation is targeted through identifying day, date, month etc. and through discussion of ADLs in relation to time and location. Attention and verbal language/fluency is targeted consistently throughout the session. Memory and perception are targeted in the tasks required to identify and categorise everyday items. Praxis and perceptual-motor abilities are targeted through the physical activity and ADL practice, with executive function skills used to sequence and plan ADL-related activities.  Completion of an ADL task is an essential component of the group, as Ryan and Dolan’s (2022) scoping review indicated CS interventions with an ADL component report benefits for ADL outcomes. | |
| **Session Number:** | 2 of 7 |
| **Group Title:** | Cognitive stimulation in activities of daily living for individuals with mild-to-moderate dementia |
| **Session Theme:** | Afternoon routine |
| **Preparation:** | |
| *Set-up* | Arrange adequate seats around a table in the centre of the room. Ensure a newspaper is available on the table. Ensure a whiteboard/flipchart and a clock are set up and visible in the room. Ensure all other materials necessary are available in the room. Set up electric bed if required with adequate space for demonstration. |
| *Materials* | Whiteboard/flipchart, marker, newspaper, inflatable ball, laminated print out of group song, plastic cups, small ball/tennis ball, electric bed, assistive devices for the bedroom e.g., bed lever, non-slip dycem mats etc. ‘How to get up from a fall’ laminated handouts. Washing basket full of an assortment of clothes, mannequins, pen and paper, whiteboards/corkboards, small plant pots, seed packets, gardening tools, soil. |
| **Format:** | |
| *Introduction* (20 minutes) | Greetings.  Warm-up activity: Pass inflatable ball/balloon around the group. Instructions provided to introduce self while holding the ball and to tell the group about their favourite time of year.  Group asked to recall group name and song chosen last week. Laminated song sheets to be handed out to group members to sing together.  Orientation of day of the week, date, month, year, season, and time to be discussed and written on whiteboard.  Discussion of news of the week. |
| *Physical activity* (15 minutes) | Gentle seated stretches to warm-up.  Physical activity to involve a game of bowling. Split group members into smaller groups and ask them to stack plastic cups into a pyramid. Group members to then take turns bowling a tennis bowl into the cups, and re-stacking when needed. |
| *Cognitive stimulation activities* (30 minutes) | Follow up from previous session with a continued discussion and reminiscence of morning routines, e.g., do you make the bed, do you like making the bed, how do you get in and out of bed etc. Discussion of tips on making the bed and demonstration of the use of assistive devices in relation to getting in and out of bed e.g., bed lever, coloured sheets, non-slip dycem mat. Group facilitators to demonstrate use of the electric bed and to provide opportunity for group members to practice use.  Group facilitators to discuss and demonstrate how to get up from a fall out of bed, providing handouts (appendix 2.3).  An assortment of clothes is to be placed on the table. Group members are to identify and discuss different types of clothes from the pile that they would wear for different occasions. Members then asked to work together to dress mannequins for certain occasions, for example, going to the beach or for a rainy day. |
| *Break* (15 minutes) | Tea/coffee and biscuits to be provided to group members. Group facilitators to encourage casual conversation amongst members. |
| *Cognitive stimulation activities* (15 minutes) | Continued discussion of afternoon routine, discussion of going out for the day, what group members need to bring with them when leaving the house. Discussion of to do lists and reminders. Group members to practice writing down what they need when leaving the house as a reminder. Group facilitators to type these lists up later and provide a printed and laminated version to group members at the next session. Whiteboards/corkboards to be introduced and group to discuss their purpose. |
| *ADL practice* (20 minutes) | Simple gardening activity: group members to be provided with small pots, gardening tools, a variety of seed packets and a bag of soil. Facilitators to assist members in gardening task. Discussion to be encouraged throughout activity. |
| *Conclusion* (5 minutes) | Group facilitators to summarise session and ask for feedback. Group to sing group song. Facilitators to remind everyone of next session. |
| **Rationale for activities chosen:**  All sessions include the components of cognitive stimulation, physical activity, and ADL practice. This session targeted the cognitive domains of orientation through the identification day, date, month etc., and through the discussion of ADLs in relation to time and location. Attention and verbal language/fluency are targeted consistently throughout the session. Memory and perception are targeted through the dressing activity requiring the identification and categorisation of everyday items. Praxis and perceptual-motor abilities are targeted through the physical activity and ADL practice, with executive function skills used in the sequencing and planning of ADL-based activities. Furthermore, written language is targeted through the completion of writing tasks. | |
| **Session Number:** | 3 of 7 |
| **Group Title:** | Cognitive stimulation in activities of daily living for individuals with mild-to-moderate dementia |
| **Session Theme:** | Domestic activities 1 |
| **Preparation:** | |
| *Set-up* | Arrange adequate seats around a table in the centre of the room. Ensure a newspaper is available on the table. Ensure a whiteboard/flipchart and a clock are set up and visible in the room. Set up a clothesline with a table and chairs nearby. Ensure all other materials necessary are available in the room. |
| *Materials* | Whiteboard/flipchart, marker, newspaper, inflatable ball/balloon, cue cards for physical activity, laminated print out of group song, music player, pen and paper, clothesline, clothes pegs, laminated instructions sheets for clothesline activity, washing basket, ironing board, iron, various domestic items e.g., washing up liquid, shoe polish and brush, colour catchers etc. Assortment of clothes and socks, variety of scented items e.g., baby powder, flowers, polish etc. Cotton pads/tissues. |
| **Format:** | |
| *Introduction* (20 minutes) | Greetings.  Warm-up activity: Pass inflatable ball/balloon around the group. Instructions provided to introduce self while holding the ball and to tell the group about their favourite holiday destination.  Group asked to recall group name and song. Laminated song sheets to be handed out to group members to sing together.  Orientation of day of the week, date, month, year, season, and time to be discussed and written on whiteboard.  Discussion of news of the week. |
| *Physical activity* (15 minutes) | Gentle seated stretches to warm-up.  Physical activity: Prepare cue cards with questions written on the back. Then take a soft ball or balloon and throw it towards a person in the group. Pick a cue card and ask the question. The person who catches the ball answers the questions. Take turns until all questions have been answered. |
| *Cognitive stimulation activities* (30 minutes) | Discussion and reminiscence of household chores, e.g., what chores do you do around the house, do you like doing these activities, what chores did you do when you were younger. Group members asked to describe and sequence domestic tasks like doing the laundry, with some members asked to write out step-by-step instructions.  A variety of household items are placed on the table e.g., shoes and shoe polish, washing up liquid, sponge, gloves, cleaning products, cloths etc. Group members are asked to discuss and demonstrate the use of these items and pair items that would be used together.  Group members to be split into smaller groups if appropriate (dependent on group size). One group to take part in a clothesline activity, the other group to take part in a matching activity. Groups will then be rotated.  Matching activity: unpaired socks are to be hidden in a pile of clothes and group members will be asked to find the socks and pair them.  Clothesline activity: laminated instruction cards stating, ‘1 pair of blue socks’, ‘3 shirts’, or a ‘red jumper’, to be used in this task (appendix 2.4). Group members asked to pick a card, read out the instruction and search for the items in the pile of clothes. Items are then to be hung up on the clothesline. Group members encouraged to work together. |
| *Break* (15 minutes) | Tea/coffee and biscuits to be provided to group members. Group facilitators to encourage casual conversation amongst members. |
| *Cognitive stimulation activities* (20 minutes) | Sensory activity: a variety of different household scents e.g., baby powder, flowers, shoe polish, washing up liquid, should be placed on cotton pads and group members are asked to identify the smells.  Discussion of evening routine e.g., what do you have for dinner, when do you have dinner. Group members to decide on a dinner to make together for next weeks session, with members writing out a menu. Group facilitators to type up, print and laminate the menu, to be used in the next session. |
| *ADL practice* (15 minutes) | Preparation of a sandwich: group members provided with a variety of sandwich ingredients and asked to prepare a sandwich of their preference. Discussion of lunch preferences to be encouraged by group facilitators. |
| *Conclusion* (5 minutes) | Group facilitators to summarise session and ask for feedback. Group to sing group song. Facilitators to remind everyone of next session. |
| **Rationale for activities chosen:**  All sessions include the components of cognitive stimulation, physical activity, and ADL practice. This session targeted the cognitive domains of orientation through the identification day, date, month etc., and through the discussion of ADLs in relation to time and location. Attention and verbal language/fluency are targeted consistently throughout the session. Memory and perception are targeted through activities requiring the identification and categorisation of everyday items/scents. Praxis and perceptual-motor abilities are targeted through the physical activity and ADL practice, with executive function skills used in the sequencing and planning of ADL-based activities. Furthermore, this task targets the domain of written language/reading and numerical capacity, through the clothesline activity. | |
| **Session Number:** | 4 of 7 |
| **Group Title:** | Cognitive stimulation in activities of daily living for individuals with mild-to-moderate dementia |
| **Session Theme:** | Evening routine |
| **Preparation:** | |
| *Set-up* | Arrange adequate seats around a table in the centre of the room. Ensure hand sanitiser and facemasks are available on the table in accordance with COVID-19 preventative measures. Ensure a newspaper is available on the table. Ensure a whiteboard/flipchart and a clock are set up and visible in the room. Set up a separate cooking station near an outlet with hotplates on the table (if a hob is not already available in the room). Ensure all other materials necessary are available in the room. |
| *Materials* | Whiteboard/flipchart, marker, newspaper, inflatable ball, laminated print out of group song, tennis ball/small ball, targets, laminated copy of dinner menu, cardboard boxes, assortment of kitchen utensils, chopping board, frying pan, tongs, peelers, knives, hotplates, dinner ingredients e.g., potatoes, carrots, pork chops, oil. An assortment of non-kitchen related items e.g., toothpaste, deodorant, hairbrush, clothes etc. Laminated charades instruction cards, tin foil, take-away plates, variety of assistive technology relating to evening routine e.g., one button radio, easy to use TV remote, informational handouts. |
| **Format:** | |
| *Introduction* (20 minutes) | Greetings.  Warm-up activity: Pass inflatable ball/balloon around the group. Instructions provided to introduce self while holding the ball and to tell the group about their favourite holiday e.g., Halloween, Christmas, easter.  Group asked to recall group name and song chosen. Laminated song sheets to be handed out to group members to sing together.  Orientation of day of the week, date, month, year, season, and time to be discussed and written on whiteboard.  Discussion of news of the week. |
| *Physical activity* (15 minutes) | Gentle seated stretches to warm-up.  Physical activity to involve a game of shooting at targets. Group members to split into smaller groups. Group facilitator to assist in setting up targets at varying distances. Group members to compete by throwing a small ball at the targets. Difficulty of targets to be adjusted as the game progresses. |
| *Cognitive stimulation activities* (20 minutes) | Recap of previous session: discuss evening routine and typical dinner routine. Present chosen menu from previous session and ask group members to discuss and identify utensils and equipment needed to prepare the dinner. A group member is to be nominated to write down a list of utensils identified.  Using the list, group members to rummage through cardboard boxes filled with a variety of items (including non-kitchen related items) and to identify and select utensils needed for the task. |
| *ADL practice* (25 minutes) | Group members to split into two smaller groups to prepare the dinner, with some members nominated to supervise the cooking of the food.  Charades activity: while food is cooking, a game of ADL-themed charades is to be played. Laminated print outs of different ADLs e.g., washing the dishes, sweeping the floor, to be presented to a group member who must act out the ADL with gestures only for the rest of the group to guess. |
| *Break* (15 minutes) | Tea/coffee and biscuits to be provided to group members. Group facilitators to encourage casual conversation amongst members. |
| *Cognitive stimulation activities* (20 minutes) | Discussion of evening routine e.g., evening activities like exercise, reading, relaxation etc. Discussion and reminiscence of TV and radio. Group facilitator to demonstrate a variety of assistive technology e.g., one button radio, easy to use TV remote, memo minder, easy use phone. Group members to be provided with the opportunity to practice use of devices, facilitators to encourage discussion of use of devices in daily life.  Informational handouts of the assistive devices to be provided. |
|  |  |
| *Conclusion* (5 minutes) | Group facilitators to summarise session and ask for feedback. Group to sing group song. Facilitators to remind everyone of next session. |
| **Rationale for activities chosen:**  All sessions include the components of cognitive stimulation, physical activity, and ADL practice. This session targeted the cognitive domains of orientation through the identification day, date, month etc., and through the discussion of ADLs in relation to time and location. Attention and verbal language/fluency are targeted consistently throughout the session. Memory and perception are targeted through activities requiring the identification and categorisation of everyday items. Praxis and perceptual-motor abilities are targeted through the physical activity, charades game and ADL practice, with executive function skills used in the sequencing and planning of ADL-based activities. Furthermore, this task targets the domain of written language through the writing task and through the reading of instructions for the charades activity. | |
| **Session Number:** | 5 of 7 |
| **Group Title:** | Cognitive stimulation in activities of daily living for individuals with mild-to-moderate dementia |
| **Session Theme:** | Domestic activities 2 |
| **Preparation:** | |
| *Set-up* | Arrange adequate seats around a table in the centre of the room. Ensure hand sanitiser and facemasks are available on the table in accordance with COVID-19 infection preventative measures. Ensure a newspaper is available on the table. Ensure a whiteboard/flipchart and a clock are set up and visible in the room. Ensure all other materials necessary are available in the room. |
| *Materials* | Whiteboard/flipchart, marker, newspaper, inflatable ball, laminated print out of group song, balloon, pen and paper, variety of coins, a variety of items bought in the grocery store e.g., eggs, milk, bread etc., laminated cards with prices for items, diaries and laminated instructional handouts, pen and paper, cardboard box, variety of kitchen utensils and non-kitchen utensils, including a juicer, pitcher, wooden spoon, cups, and knives. Lemons, sugar, and water. |
| **Format:** | |
| *Introduction* (20 minutes) | Greetings.  Warm-up activity: Pass inflatable ball/balloon around the group. Instructions provided to introduce self while holding the ball and to tell the group a story about where they are from.  Group asked to recall group name and song. Laminated song sheets to be handed out to group members to sing together.  Orientation of day of the week, date, month, year, season, and time to be discussed and written on whiteboard.  Discussion of news of the week. |
| *Physical activity* (15 minutes) | Gentle seated stretches to warm-up.  Physical activity to involve a game of keeping a balloon in the air. A balloon is to be passed around the group with the intention of keeping it in the air and not letting it fall to the ground. The final group member must pass the ball into a goal. If the ball falls or misses the target the game restarts. |
| *Cognitive stimulation activities* (30 minutes) | Discussion of grocery shopping e.g., how do you get to the shops? Do you drive, walk, use public transport? What shops do you go to? What do you usually buy?  Discuss price of shopping, discuss old currencies e.g., pennies and shillings.  Group members to write out a typical grocery list. Group to discuss and guess prices for certain items and discuss what they might have cost in the old currency.  Numbers-based grocery activity: Members are to split into smaller groups. Group members are to be provided with a variety of coins: 5c, 10c, 50c, 1 euro etc. A variety of typical items bought in the grocery shop e.g., butter, eggs, milk, to be placed on the table. Laminated cards (‘price tags’) with the name of the item and its corresponding price are to be placed in a pile (appendix 2.5). Pile of laminated ‘price tags’ should also include items that are not available on the table in order to promote group members recognition and categorisation abilities. Group members are to work together to find the price for the item and must add up their coins to the correct amount for the item. A pen and paper to be provided by the group facilitators if needed. At the end, group members should add up the total amount for the items. |
| *Break* (15 minutes) | Tea/coffee and biscuits to be provided to group members. Group facilitators to encourage casual conversation amongst members. |
| *Cognitive stimulation activities* (25 minutes) | Continued discussion of shopping, discuss using lists, reminders. Introduce diaries and provide handouts. Discuss uses of the diary e.g., writing out to-do list for grocery shopping.  Discussion of old recipes for fruit punch, including method and ingredients. Discussion and reminiscence of lemonade recipes, nominate one member of the group to write out a recipe. If participants are unable to recall a recipe, provide prompts or provide a pre-printed recipe.  Group members to rummage through a cardboard box and identify and categorise ingredients and utensils used to make lemonade. |
| *ADL practice* (20 minutes) | Members to split into smaller groups and work together to make lemonade. Group facilitators to encourage discussion throughout. |
| *Conclusion* (5 minutes) | Group facilitators to summarise session and ask for feedback. Group to sing group song. Facilitators to remind everyone of next session. |
| **Rationale for activities chosen:**  All sessions include the components of cognitive stimulation, physical activity, and ADL practice. This session targeted the cognitive domains of orientation through the identification day, date, month etc., and through the discussion of ADLs in relation to time and location. Attention and verbal language/fluency are targeted consistently throughout the session. Memory and perception are targeted through activities requiring the identification and categorisation of everyday items. Praxis and perceptual-motor abilities are targeted through the physical activity and ADL practice, with executive function skills used in the sequencing and planning of ADL-based activities. Furthermore, this task targets the domain of written language through the writing task and through the reading of instructions for the numbers-based activity. Numerical capacity and calculation are targeted through this activity. | |
| **Session Number:** | 6 of 7 |
| **Group Title:** | Cognitive stimulation in activities of daily living for individuals with mild-to-moderate dementia |
| **Session Theme:** | Baking |
| **Preparation:** | |
| *Set-up* | Arrange adequate seats around a table in the centre of the room. Ensure hand sanitiser and facemasks are available on the table in accordance with COVID-19 preventative measures. Ensure a newspaper is available on the table. Ensure a whiteboard/flipchart and a clock are set up and visible in the room. Ensure kitchen staff are aware of need to use oven. Ensure all other materials necessary are available in the room. |
| *Materials* | Whiteboard/flipchart, marker, newspaper, inflatable ball, laminated print out of group song, bean bag, floor targets, cookie ingredients, e.g., butter, eggs, sugar, flour, chocolate chips. Baking utensils e.g., mixing bowls, stand/handheld mixer, wooden spoons, baking tray, greaseproof paper, weighing scales. Laminated printouts of recipe, pen and paper, timer, wash basins, towels, sponge, washing up liquid, sweeping brush, disinfectant spray, wipes, takeaway plates and tinfoil. |
| **Format:** | |
| *Introduction* (20 minutes) | Greetings.  Warm-up activity: Pass inflatable ball/balloon around the group. Instructions provided to introduce self while holding the ball and to tell the group about what type of food they eat during the current season e.g., summer.  Group asked to recall group name and song. Laminated song sheets to be handed out to group members to sing together.  Orientation of day of the week, date, month, year, season, and time to be discussed and written on whiteboard.  Discussion of news of the week. |
| *Physical activity* (15 minutes) | Gentle seated stretches to warm-up.  Physical game to involve bean bag toss. Targets with different points allocated to each target to be placed on the floor. Group members to be split into teams and encouraged to compete against each other by aiming to score the most points. The whiteboard can be used to record points for each team. |
| *Cognitive stimulation activities* (20 minutes) | Discussion and reminiscence of baking recipes, including recipes from childhood. Group members asked to discuss recipes for cookies and write down ingredients/sequencing of recipe. If members do not recall a recipe, prompts or a laminated print out of a cookie recipe should be provided.  Group members are to split into smaller teams. A variety of baking utensils and non-baking items are to be placed on the table. Group members are asked to identify items they will need for the task, discussing and demonstrating the use of the utensil, e.g., handheld mixer. |
| *ADL practice* (25 minutes) | Group members will work together in their teams to follow the recipe and make cookies. Group facilitators will assist group members in weighing out ingredients. Once cookies have been prepared, facilitators should place them in the kitchen oven if available. |
| *Break* (15 minutes) | Tea/coffee and biscuits to be provided to group members. Group facilitators to encourage casual conversation amongst members. |
| *ADL practice* (20 minutes) | Cleaning activity: group members to be asked to assist in cleaning up after the baking activity. Wash basins, towels, washing up liquid, sweeping brushes, sponges and wipes should be provided, and group members should work together to clean the dishes and table. Group facilitators should provide assistance where appropriate. Discussion of household chores should be encouraged throughout this task, e.g., use of a dishwasher, do you like cleaning the dishes.  Group facilitators should take cookies out of the oven and encourage group members to serve up the items. |
| *Conclusion* (5 minutes) | Group facilitators to summarise session and ask for feedback. Group to sing group song. Facilitators to remind everyone of next session. |
| **Rationale for activities chosen:**  All sessions include the components of cognitive stimulation, physical activity, and ADL practice. This session targeted the cognitive domains of orientation through the identification of day, date, month etc., and through the discussion of ADLs in relation to time and location. Attention and verbal language/fluency are targeted consistently throughout the session. Memory and perception are targeted through activities requiring the identification and categorisation of everyday items. Praxis and perceptual-motor abilities are targeted through the physical activity and ADL practice, with executive function skills used in the sequencing and planning of ADL-based activities. Furthermore, this task targets the domain of written language through the writing task and reading of recipe instructions and through the reading of instructions. Numerical capacity is also targeted through the weighing of ingredients. | |
| **Session Number:** | 7 of 7 |
| **Group Title:** | Cognitive stimulation in activities of daily living for individuals with mild-to-moderate dementia |
| **Session Theme:** | Entertainment and leisure |
| **Preparation:** | |
| *Set-up* | Arrange adequate seats around a table in the centre of the room. Ensure hand sanitiser and facemasks are available on the table in accordance with COVID-19 infection control advice. Ensure a newspaper is available on the table. Ensure a whiteboard/flipchart and a clock are set up and visible in the room. Ensure all other materials necessary are available in the room. |
| *Materials* | Whiteboard/flipchart, marker, newspaper, inflatable ball, laminated print out of group song, CD player/speaker, a variety of CDs, projector, laptop, presentation including photos of musicians/showbands with links to music videos, a variety of leisure items e.g., knitting needles, wool, cards, dominoes, word puzzles, books, jigsaws, books, musical instruments. Laminated print outs on colured card of line-dancing instructions. |
| **Format:** | |
| *Introduction* (20 minutes) | Greetings.  Warm-up activity: Pass inflatable ball/balloon around the group. Instructions provided to introduce self while holding the ball and to tell the group what they like to do in their free time.  Group asked to recall group name and song. Laminated song sheets to be handed out to group members to sing together.  Orientation of day of the week, date, month, year, season, and time to be discussed and written on whiteboard.  Discussion of news of the week. |
| *Physical activity* (15 minutes) | Gentle seated stretches to warm-up.  Physical activity to involve a game of skittles. |
| *Cognitive stimulation activities* (25 minutes) | Discussion of favourite past-times and leisure activities. Discussion and reminiscence of music, dance halls and different dance routines.  A variety of CDs to be placed on the table and group members encouraged to pick and discuss CDs they recognise. Group members to choose music to be played. Group facilitators to present presentation of showbands to group members and encourage members to guess their names, providing hints through music. Presentation to include photos of local dance halls, outfits etc. Discussion of price of tickets to see showbands etc.  Group members chairs are to be placed in a line, with lead group facilitator in the middle. Laminated print outs on coloured card of line-dancing instructions to be placed in front of each group members (appendix 2.6). Group facilitator to demonstrate seated line-dancing in beat with music of choice. |
| *Break* (15 minutes) | Tea/coffee and biscuits to be provided to group members. Group facilitators to encourage casual conversation amongst members. |
| *Cognitive stimulation activities* (15 minutes) | A variety of leisure items are to be placed on the table, e.g., jigsaws, word puzzles, knitting needles, dominoes, cards, musical instruments. Group members encouraged to pick an item they enjoy and demonstrate to the group how to use it if appropriate e.g., knitting, card games, musical instruments. |
| *ADL practice* (25 minutes) | Group members encouraged to split into smaller groups and take part in a leisure activity of their choice, e.g., a card game, dominoes, knitting. |
| *Conclusion* (5 minutes) | Group facilitators to summarise session and ask for feedback. Group to sing group song. Facilitators to conclude group and thank everyone for their attendance. Remind everyone that this is the last session. |
| **Rationale for activities chosen:**  All sessions include the components of cognitive stimulation, physical activity, and ADL practice. This session targeted the cognitive domains of orientation through the identification of day, date, month etc., and through the discussion of leisure activities in relation to time and location. Attention and verbal language/fluency are targeted consistently throughout the session. Memory is targeted through the discussion and reminiscence of music, showbands, and leisure activities. Perception is targeted through the completion of activities like card games, jigsaws etc. Praxis and perceptual-motor abilities are targeted through the physical activity and completion of leisure activities like knitting, jigsaws, and use of musical instruments. Written language is also targeted through the presentation of CDs and showbands. | |

*Appendix 2.2 Morning routine category instruction cards*

| Breakfast |
| --- |
| Getting ready for the day |
| Cleaning |

Instruction cards to be printed on A4 coloured paper and laminated.

*Appendix 2.3 How to get up from a fall instructional handout*

| **What to do if you fall**  If you fall, try to stay calm. Take time to assess the situation as it can take a few minutes to feel pain from injuries. What you do next will depend on if you’re hurt and whether or not you’re able to get up without help.  **Checking for injuries**  The first thing you need to do after a fall is work out if you’re hurt. Take a few minutes to check your body for any pain or injuries, then:   - If you’re not hurt, try to get up from the floor - If you’re hurt or unable to get off the floor, call for help and keep warm and moving as best you can while you wait.   **Getting up from a fall**  If you’re not hurt and feel well enough, you could try to get up from the floor. The best way will differ from person to person, but as a guide, you can:   1. Roll onto your side 2. Push yourself up to a side sitting position 3. Slowly get onto your hands and knees 4. Crawl towards a sturdy piece of furniture that can support you to get up- such as a solid chair or sofa. 5. Kneel side-on to the chair or sofa with your strongest leg next to it, then slide the foot of your strongest leg forward so that it’s flat on the floor. Your other knee should remain on the floor. 6. Put both hands on the chair or sofa. 7. Raise and turn your body, pushing through your hands and foot until your bottom is safely on the chair or sofa. 8. Sit for a few minutes before you try to do anything else and check again for injuries.   If you have weak muscles, or painful and stiff joints in your legs, you may not be able to follow these instructions. In this case, you can consider getting a pendant alarm so you know that help will always be available.To improve your confidence and technique, it’s a good idea to practice getting up from a fall in different rooms of your house and using different types of sturdy furniture for support. For your safety, ask a friend or relative to be with you when you practice. |
| --- |

Handouts to be printed on A4 paper.

*Appendix 2.4 Clothesline activity sample instruction cards*

| An orange jumper | 2 pairs of green socks | 3 shirts |
| --- | --- | --- |

Instruction cards to be printed on A4 coloured paper and laminated.

*Appendix 2.5 Numbers-based grocery activity sample instruction cards*

| €1.00 - Milk | €2.79 - Butter | €1.99 - Eggs | €2.50 - Cheese |
| --- | --- | --- | --- |

Instruction cards to be printed on individual, coloured strips of paper and laminated.

*Appendix 2.6ap Line-dancing instruction cards*

| Heels in and out x 4  Right leg out and in x2  Left leg out and in x2  Slap legs, hands to shoulder x 4  Shimmy forward and back x2  REPEAT! |
| --- |

Instruction cards to be printed on coloured A4 paper and laminated.
