# Supplementary material for: Cognitive stimulation in activities of daily living for individuals with mild-to-moderate dementia (CS-ADL): Study protocol for a randomised controlled trial
Source: PLoS One. 2024 Sep 3;19(9):e0309337. doi: 10.1371/journal.pone.0309337 (PMC11371241; doi:10.1371/journal.pone.0309337)
Supplement: Attachment — Submitted filename: Rebuttal letter.docx [file pone.0309337.s004.docx]

| **Query/prompt** | **Author response** |
| --- | --- |
| a) If there are ethical or legal restrictions on sharing a de-identified data set, please explain them in detail (e.g., data contain potentially identifying or sensitive patient information, data are owned by a third-party organization, etc.) and who has imposed them (e.g., a Research Ethics Committee or Institutional Review Board, etc.). Please also provide contact information for a data access committee, ethics committee, or other institutional body to which data requests may be sent. | There are ethical restrictions placed on sharing a de-identified data set from this study, as data includes sensitive, personally identifiable patient information that would be unethical to release without anonymisation. This requirement has been imposed by the General Data Protection Regulation (GDPR) in Ireland, the Irish Data Protection Act (2018) and the Research Ethics Committees that adhere to these regulations that approval was sought from for this study. As specified in the manuscript, all relevant, anonymised data produced from this study will be made publicly available upon study completion via an online repository (e.g., Figshare). If you wish to contact the ethics committees for further clarification, their contact details are below:  North East REC: [NorthEast.REC@hse.ie](mailto:northEast.REC@hse.ie)  Galway Clinical REC: [colette.collins@hse.ie](mailto:colette.collins@hse.ie)  Midlands REC: [REC.B.CorporateMidlands@hse.ie](mailto:REC.B.CorporateMidlands@hse.ie) |
| b) If there are no restrictions, please upload the minimal anonymized data set necessary to replicate your study findings to a stable, public repository and provide us with the relevant URLs, DOIs, or accession numbers. | As this is a protocol, there are no datasets currently available to share. As stated in the manuscript, a fully anonymised dataset will be made publicly available upon study completion via an online repository (e.g. Figshare). |
| 3. Your ethics statement should only appear in the Methods section of your manuscript. If your ethics statement is written in any section besides the Methods, please move it to the Methods section and delete it from any other section. Please ensure that your ethics statement is included in your manuscript, as the ethics statement entered into the online submission form will not be published alongside your manuscript. | The ethics statement appears in the Methods section of the manuscript only under ‘ethical considerations and trial registration’ in the Materials and Methods section. |
| 4. Please include captions for your Supporting Information files at the end of your manuscript, and update any in-text citations to match accordingly. | Captions have been provided accordingly for supporting information at the end of the manuscript. |
| **Reviewer Comments** | **Author responses** |
| 1. Why a medium effect size (d=0.5) is chosen, please explain the rationale behind it. | As specified in the ‘trial design’ subheading of the ‘Materials and Methods’ heading, while ideally a large effect size (*d=0.2)* is preferable, this would equate to a sample size of 199. It will be expected there will be challenges in the recruitment of 199 participants for the scale and timeframe of this study (approx. 1 year), and due to the challenges noted in recruiting this population in the literature (Langbaum et al., 2023). Therefore, for pragmatic reasons, recruitment will aim for the sample size of 34 (*d=0.5).* |
| 2. Two primary outcome is chosen BADL and IADL. This will require multiple testing adjustment s otherwise type-1 error will be inflated. This is not addressed. | Thank you for your comment, this is an error on my part. I have adjusted the primary outcome to be ADL performance as opposed to both BADL and IADL, as multiple testing adjustment is not planned. |
| 3. How the missing data will be handled? | It is possible that some data will be missing. To avoid this, attempts to follow-up with all participants will be made. However, if missing data remain, intention-to-treat analysis will be used to account for this. |
| 4. The power analysis is based on two-sample t-tests, but the Statistical analysis section talks about regression-based model. They are not exactly the same, model for power analysis and primary analysis model should be as close possible as otherwise power analysis will be not be that meaningful. | Thank you for this advice, appropriate revisions have been made in text. Types of tests used in analysis will be chosen following completion of the dataset to assess whether the data is parametric or non-parametric. |
| This is clearly a complex intervention and therefore I wonder why the authors do not take the UK MRC framework for the development and evaluation of complex interventions into account. They do not report a programme theory or a logic model, do not consider a process evaluation and an economic evaluation. I wonder whether it is still timely to disregard the MRC framework, which has undoubtedly proven as golden standard. | Discussion of the MRC framework has been applied to the development of CS-ADL. A process evaluation and an economic evaluation are outside the scope of this study and will be focused on in future research. |
| The hypotheses passage is unnessessary and reads like a textbook information. | Thank you for your recommendation, this section has been removed from the manuscript. |
| The sample size is rather low and I wonder whether the estimated effact size could be translated to a minimum clinical important difference. Apparently, a drop out rate has not been taken into account. | Thank you for this suggestion. In line with this suggestion, it has been included in text that the minimum clinically important difference will be calculated based on the medium effect size, by dividing the difference in scores from baseline to posttreatment by the standard deviation of baseline scores (Franceschini et al. 2023).  In relation to the consideration of a drop-out rate, the following has been inserted in text: While this study is of a short duration, it is anticipated that there will be a minimum 10% attrition rate. This figure stems from Ritchie, Gillen & Grill’s (2023) analysis of dementia trial retention data. Therefore, recruitment will aim to exceed the estimated sample size by 10% to ensure the study is adequately powered. |
| The authors do not completely adhere to the reporting statement. It remains unclear who will be in charge of the implementation of the allocation sequence and how this will be done. | Random allocation will be completed by one of the principal investigator’s academic supervisors who are otherwise uninvolved in the conduct of the study |
| Where will the intervention take place? Please add information. | Intervention will take place at the clinical sites where recruitment will take place. CS-ADL groups will be facilitated in available group therapy/ occupational therapy rooms available. |
| Do the authors plan to implement a control mechanism whether blinding was successful? | The success of blinding will be tested following completion of outcome measures by asking assessors to guess the treatment assignment of participants, expressing either a treatment guess or uncertainty. If blinding is successful, ‘do not know’ guesses should be high, and incorrect guesses should be balanced (Kolahi, Bang & Park, 2009). While it is possible that unblinding might occur, i.e., outcome assessors become aware of group allocation, the process of blinding will be explained to participants, and they will be asked to not reveal their treatment allocation to the assessor. |
| A figure displaying the participant timelime is missing. | Apologies for this error. The figure in question has been uploaded with this resubmission. |
| It remains unclear why an audit is not planned. Please comment on this. An audit would guarantee the quality of study procedures and data. | While it is acknowledged that an audit would ensure the quality of study processes, traditional auditing methods are expensive and due to constraints on budget and resources for this study, a formal audit process will not be completed. The researcher will instead attend carefully to the research design throughout the process of the study. Furthermore, careful thought and consideration has gone into the planning of the pragmatics of the study to ensure processes are feasible within the clinical settings in which the study is run, to minimise deviations from the protocol. |
